# Interferon-γ Response of *Mycobacterium avium* subsp. *paratuberculosis* Infected Goats to Recombinant and Synthetic Mycobacterial Antigens

**DOI:** 10.3389/fvets.2021.645251

**Published:** 2021-03-26

**Authors:** Heike Köhler, Elisabeth Liebler-Tenorio, Valerie Hughes, Karen Stevenson, Douwe Bakker, Peter Willemsen, Sylvie Bay, Christelle Ganneau, Franck Biet, H. Martin Vordermeier

**Affiliations:** ^1^National Reference Laboratory for Paratuberculosis, Institute of Molecular Pathogenesis, Friedrich-Loeffler-Institut, Jena, Germany; ^2^Institute of Molecular Pathogenesis, Friedrich-Loeffler-Institut, Jena, Germany; ^3^Vaccines and Diagnostics Department, Moredun Research Institute, Penicuik, United Kingdom; ^4^Department of Infection Biology, Wageningen Bioveterinary Research, Lelystad, Netherlands; ^5^Unité de Chimie des Biomolécules, Département de Biologie Structurale et Chimie, Institut Pasteur, Paris, France; ^6^CNRS UMR 3523, Paris, France; ^7^INRAE, Université de Tours, ISP, Nouzilly, France; ^8^Animal and Plant Health Agency, Addlestone, United Kingdom

**Keywords:** experimental infection, goat, IFN-gamma response, *Mycobacterium avium subsp. hominissuis* (MAH), *Mycobacterium avium subsp. paratuberculosis* (MAP), recombinant proteins, synthetic lipopetides

## Abstract

Despite its potential for early diagnosis of *Mycobacterium avium* subsp. *paratuberculosis* (MAP) infection, the IFN-γ release assay is not used routinely, because of low specificity of the established crude antigen preparation Johnin (PPDj). Limited data are available assessing the potential of MAP-derived protein and lipopeptide antigens to replace PPDj in assays for goats, while cattle and sheep have been studied more extensively. Furthermore, MAP infection is claimed to interfere with the diagnosis of bovine tuberculosis when other crude antigen preparations (PPDb, PPDa) are applied. In this study, the diagnostic potential of MAP-derived recombinant protein antigens, synthetic MAP lipopentapeptides and of *Mycobacterium bovis-*specific peptide cocktails was assessed compared to crude mycobacterial antigen preparations in experimentally infected goats. Goats were inoculated with MAP, or *Mycobacterium avium* subsp. *hominissuis* (MAH) as surrogate for environmental mycobacteria, non-exposed animals served as controls. *Mycobacterium avium* Complex-specific antibody and PPDj-induced IFN-γ responses were monitored *in vivo*. Infection status was assessed by pathomorphological findings and bacteriological tissue culture at necropsy 1 year after inoculation. The IFN-γ response to 13 recombinant protein antigens of MAP, two synthetic MAP lipopentapeptides and three recombinant peptide cocktails of *Mycobacterium bovis* was investigated at three defined time points after infection. At necropsy, MAP or MAH infection was confirmed in all inoculated goats, no signs of infection were found in the controls. Antibody formation was first detected 3–6 weeks post infection (wpi) in MAH-inoculated and 11–14 wpi in the MAP-inoculated goats. Maximum PPDj-induced IFN-γ levels in MAH and MAP exposed animals were recorded 3–6 and 23–26 wpi, respectively. Positive responses continued with large individual variation. Antigens Map 0210c, Map 1693c, Map 2020, Map 3651cT(it), and Map 3651c stimulated increased whole blood IFN-γ levels in several MAP-inoculated goats compared to MAH inoculated and control animals. These IFN-γ levels correlated with the intensity of the PPDj-induced responses. The two synthetic lipopentapeptides and the other MAP-derived protein antigens had no discriminatory potential. Stimulation with *Mycobacterium bovis* peptide cocktails ESAT6-CFP10, Rv3020c, and Rv3615c did not elicit IFN-γ production. Further work is required to investigate if test sensitivity will increase when mixtures of the MAP-derived protein antigens are applied.

## Introduction

Paratuberculosis (paraTb) is one of the economically most important infectious diseases of domestic ruminants including goats (1). This deserves attention, since goats are of growing importance for human nutrition worldwide. Data of the FAO^1^ indicate that the numbers of domestic goats more than tripled globally during the last 60 years, with the most pronounced increase in Africa and Asia (2). Caused by *Mycobacterium avium* subsp. *paratuberculosis* (MAP), paraTb is a chronic progressive granulomatous enteritis resulting in malnutrition, reduction in milk yield, weight loss, and eventually death. Infection with MAP occurs in young goats and may remain clinically non-apparent for several years until clinical signs are observed. Transient episodes of shedding of MAP occur during the subclinical phase. Early diagnosis of potential shedders is of paramount importance to prevent environmental contamination and protect herds from infection. To date, diagnosis of MAP infection in living animals relies on direct methods, such as culturing MAP from feces or molecular biological detection of MAP by PCR, or indirect methods such as antibody detection in blood serum or milk by ELISA (3, 4). However, using these methods, identification of MAP infection can only be achieved years after the initial infection (5). Early diagnosis is possible by assessment of the antigen-induced interferon-γ (IFN-γ) response of blood T-cells by the IFN-γ release assay (IGRA) (6–8). Diagnostic sensitivity and specificity of the IGRA depend on the antigen preparations used for stimulation of IFN-γ production. Strong responses, i.e., high sensitivity, were elicited by crude preparations of mycobacteria-purified protein derivatives (PPDs). Johnin (PPDj) is prepared from MAP and has a variable proteomic composition containing common mycobacterial immunogenic proteins (9), which can cross-react causing false-positive results and low specificity (10, 11).

During recent years, several recombinant proteins or peptides derived from MAP were evaluated in experimental and field studies using cattle and sheep in an attempt to replace PPDj with more specific antigens (12–18). Promising protein antigen candidates have been identified, for example Map 3651c and Map 0268c. However, their immunogenicity seems to differ between host species. Furthermore, a lipopentapeptide unique to MAP of subtype C, L5P (19), proved to be immunogenic in cattle (11), while an analogous preparation, Para-LP-01, did not elicit a distinct IFN-γ response in sheep (20). Only limited data are available for goats. In one study, 10 MAP recombinant proteins were suggested as potential candidates for early detection of MAP-infected animals, and combining Map 3527, Map 2020, Map 3651c, Map 1050c, Map 0210c, and Map 4000c was recommended to optimize the sensitivity of a caprine MAP IGRA (21). However, most of these antigens also induced IFN-γ release from cells of goats classified as non-infected, which raises doubts regarding their specificity.

Bovine tuberculosis (bTb), caused by *Mycobacterium bovis*, also has a subclinical phase, during which early and sensitive diagnosis can also be performed by IGRA (22). The conventional reagents currently applied in IGRA tests for the ante-mortem diagnosis of bTb are obtained from *M. bovis* (PPDb) and *M. avium* subsp. *avium* (PPDa). The components of the PPD's are poorly characterized, difficult to standardize and not entirely specific. To overcome this issue, the diagnostic potential of antigens specific to the *M. tuberculosis* complex (ESAT-6, CFP-10, Rv3615c) was assessed and showed promising results (23, 24). The performance of these antigens when testing MAP-infected animals for bTb is not known and has to be addressed, because MAP infection impacts the specificity of the conventional IGRA for bTb (25), leading to false positive responses.

In the present study, the potential of 13 recombinant protein antigens of MAP, two synthetic MAP lipopentapeptides and three recombinant peptide antigens of *M. bovis* to induce IFN-γ production of blood cells was assessed in goats in comparison to the three PPDs. The animals were experimentally infected either with MAP, the causative agent of paraTb or with *M. avium* subsp. *hominissuis* (MAH), a closely related organism widely distributed in the environment. Uninfected animals served as controls. The aims were to further evaluate the diagnostic potential of antigen candidates for MAP, to identify sources for false positive IGRA responses with MAH as a surrogate for environmental mycobacteria and to check whether antigens used for bTb diagnosis do not elicit positive IGRA responses in MAP-infected goats.

## Materials and Methods

### Legislation and Ethical Approval

This study was carried out in strict accordance with European and National Law for the Care and Use of Animals. The protocol was reviewed by the Committee on the Ethics of Animal Experiments of the State of Thuringia, Germany, and approved by the competent authority (Permit Number: 22-2684-04-04-002/12, date of permission 12.12.2012). All experiments were done in containment of biosafety level 2 under supervision of the authorized institutional Agent for Animal Protection. During the entire study, every effort was made to minimize suffering.

### Animals

Altogether 52 goat kids of the breed: “Thüringer Wald Ziege” (47 male, two female and three hermaphrodites) were included in the study. Animals originated from a conventionally raised herd of dairy goats with no history of clinical paraTb. The adult goats of the herd were tested for the presence of MAP by fecal culture, 5 years prior to this experiment, and were found to be negative. Furthermore, 2 and 4 years before the present study, tissues (including ileum, jejunum, ileo-caecal valve, mesenteric lymph nodes, liver, hepatic lymph nodes) from 30, 1-year-old male goats from the same herd were cultured to determine if MAP was present. These were also found to be negative.

Clinically healthy goat kids aged 8–19 days and weighing between 1.9 and 4.4 kg (3.23 ± 0.67 kg; mean ± SD) were transferred to the animal facility of the Friedrich-Loeffler-Institut in Jena. The goats were allocated to three different groups (MAP-inoculated: *n* = 21, MAH-inoculated: *n* = 21, controls: *n* = 10) based on weight at birth, age, sire, and dam in order to prevent full siblings in the same group, get an equal distribution of offspring of the same sire between groups and adjust mean body weight and mean age of the groups. Throughout the entire study, the different groups were kept in separate rooms with identical housing and feeding conditions.

Feeding was adjusted to the age-dependent nutritional needs of the animals. The kids received commercial milk replacer for goat kids (Denkamilk capritop, Denkavit, Warendorf, Germany) up to the age of 10 weeks. Water, mineral blocks without copper, containing 37% sodium, 1.1% calcium, 0.6% magnesium, and trace elements (Mineralleckstein ohne Kupfer, esco—european salt company, Hannover, Germany) and hay were supplied *ad libitum* during the whole course of the experiment. Small amounts of pelleted concentrates were offered during milk feeding. After weaning, protein-rich pelleted concentrates for goats (Alleinfuttermittel für Ziegenmastlämmer and Milchleistungsfutter II, both LHG Landhandelsgesellschaft, Schmölln, Germany) containing vitamin A, D3, and E were fed. The ration of concentrates was gradually increased up to 500 g per day. None of the feed contained antibiotics. The male animals were castrated at ~12 weeks of age. Throughout the experiment, the animals were not treated with systemic antibiotics or anti-inflammatory drugs.

### Preparation of MAP and MAH Used for Inoculation

A low passage C-type MAP field isolate from the jejunum of a cow (JII-1961) (26) and a MAH field isolate from a mesenteric lymph node of a naturally infected pig (09MA1289) (27) were used for inoculation. The bacterial stocks were prepared essentially as described previously (28). In short, the isolates were propagated in Middlebrook 7H9 broth with glycerol 0.2%v/v, OADC 10%v/v, and Mycobactin J 0.2%v/v at 37 ± 2°C. No antibiotics were added. Different parallel culture batches were pooled and then dispensed to form the bacterial inoculum stocks. After centrifugation of the stocks, the bacterial wet mass (bwm) of each pellet was determined. The stocks were stored at 5 ± 3°C. One stock was used per inoculation day. Two to three days before inoculation the pellet was re-suspended to a concentration of 1 mg bwm/mL using phosphate buffered saline (PBS). This batch suspension was incubated at 37 ± 2°C until the day of inoculation. Then, 10 mL of the suspension were dispensed into separate tubes to form the inoculum for each individual animal. Bacterial counts of the respective batch suspension were determined as described elsewhere (28). The inoculum amounted to 6.28 ± 4.07 × 10^7^ cfu/dose for MAP and to 2.29 ± 0.99 × 10^9^ cfu/dose for MAH. For further characterization of the batch suspensions, acid-fast bacilli (AFB) were confirmed by Ziehl-Neelsen staining, MAP was confirmed by IS*900* PCR using primers according to Englund et al. (29) and MAH was confirmed by PCR proving presence of IS*1245* (30) and absence of IS*901* (31). Freedom from contaminating bacteria was tested by inoculation on blood agar plates.

### Study Design

One group of goats was challenged with MAP (*n* = 21), one group with MAH (*n* = 21), and 10 animals served as controls. Oral inoculation started 10–21 days post natum and was performed 10 times every 2–4 days. Each individual bacterial dose (10 mg bwm) was suspended in 50 mL of pre-warmed milk replacer in a baby bottle. The goats were bottle-fed with the inoculum prior to regular morning feeding. Control animals received the same amount of pure milk replacer.

At the beginning of the experiment, eight randomly selected animals from each group were allocated to subgroups for antigen testing. All animals were used for other studies (27, 32).

**Figure 1:**
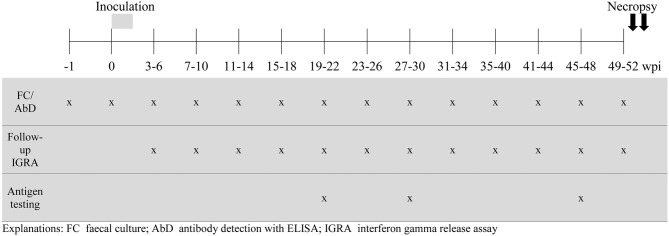
During the course of the experiment, fecal samples and blood were collected 1 week before the start of the inoculation period (−1 wpi), directly before the first inoculation (0 wpi) and then in regular intervals until 49–52 wpi. The goats were divided into four sampling subgroups, consisting of 2 animals of each treatment group, that were sampled 7 days apart such that all animals were sampled once every 4 weeks. The results of one 4-week period were taken together to form one time period for analysis.

Each goat underwent daily clinical examination from the beginning of the experiment until necropsy. Parameters to follow the course of infection were: fecal shedding of MAP; serum antibody response against MAP and MAH and specific whole blood IFN-γ response. Individual blood and fecal samples were collected to examine humoral immune response and fecal shedding of MAP or MAH before the first inoculation and in regular intervals after inoculation. The amount of blood that could be collected from very young goat kids was limited because of animal welfare reasons. Therefore, testing of the cellular immune response started at 3–6 wpi and continued until necropsy (Figure 1). The whole blood IFN-γ response to the test antigens was examined at three different time points, 19–22, 27–30, and 45–48 wpi.

Goats were euthanized and necropsied about 12 months after the last inoculation. Gross and histologic lesions and bacterial culture positive tissues were recorded.

### Antigens

Antigens were either purchased from commercial suppliers or prepared essentially as described elsewhere (see Table 1). PPDj was provided by Douwe Bakker, CVI-WUR, The Netherlands.

**Table 1:** IGRA: Designation and kind of antigens used for whole blood stimulation, final concentrations applied and their source including reference.

**Antigen**	**Kind of antigen**	**Putative function**	**Final concentration**	**References**
PWM^a^	Mitogen		5 μg/mL	
Johnin (PPDj, MAP PPD)^b^	Antigen preparation from culture material		5 μg/mL	
PPDa (M. *avium avium* PPD)^c^	Antigen preparation from culture material		125 U/mL	
PPDa (M. *avium avium* PPD)^a^	Antigen preparation from culture material		250 U/mL	
PPDb (M. *bovis* PPD)^a^	Antigen preparation from culture material		300 U/mL	
ESAT6-CFP10 (MTC)^d^	Peptide cocktail	Secreted antigens	5 μg/mL each peptide	(24)
Rv3615c (MTC)^d^	Peptide cocktail	EspC, ESX-1 secretion-associated protein	5 μg/mL each peptide	(24)
Rv3020c (MTC)^d^	Peptide cocktail	EsxS, ESAT-6 like protein	5 μg/mL each peptide	(33)
L5P (MAP)^e^	Synthetic lipopeptide	Lipopentapeptide major cell wall lipopeptide	10/20 μg/mL	(19, 34, 35)
Hydrosoluble (Hyd) L5P (MAP)^e^	Synthetic lipopeptide	As above	10/20 μg/mL	(19, 34, 35)
Map 0268c^f^	Protein	Putative Thiopurine S-methyltransferase	10/20 μg/mL	(16)
Map 0268c(it)^*^^f^	Protein	As above	10/20 μg/mL	(16)
Map 1365^f^	Protein	ArgF ornithine carbamoyl transferase	10/20 μg/mL	(16)
Map 3651cT(it)^*^^f^	Truncated protein	FadE3_2, Acyl-CoA dehydrogenase	10/20 μg/mL	(16)
Map 4147^f^	Protein	Putative ferredoxin reductase	10/20 μg/mL	(16)
Map 2872c^f^	Protein	FabG5_2	10/20 μg/mL	(16)
Map 1589c^b^	Protein	AhpC, Alkyl hydroperoxide reductase C	10/20 μg/mL	(21, 36)
Map 1653^b^	Protein	Tpx, putative thiol peroxidase	10/20 μg/mL	(21, 36)
Map 3651c^b^	Protein	As above	10/20 μg/mL	(21, 36)
Map 1693c^b^	Protein	Peptidyl-propyl cis-trans isomerase	10/20 μg/mL	(21, 36)
Map 0210c^b^	Protein	Secreted PirG protein	10/20 μg/mL	(21, 36)
Map 4000c^b^	Protein	Putative ESAT-6 like protein CFP10	10/20 μg/mL	(21, 36)
Map 2020^b^	Protein	Putative hydrolase	10/20 μg/mL	(21, 36)

* *It isopropanol treated*.

*Source of antigen*:

a *Prionics*;

b *CVI-WUR Central Veterinary Institute-Wageningen University & Research, Lelystad, The Netherlands*;

c *WdT Wirtschaftsgenossenschaft deutscher Tierärzte eG, Garbsen, Germany*;

d *APHA Animal and Plant Health Agency, Addlestone, United Kingdom*;

e *IP Institute Pasteur, Paris, France*;

f *MRI Moredun Research Institute, Penicuik, United Kingdom*.

*Gene annotation (GenBank AE016958) according to Li et al. (37) and putative function sourced from KEGG (38)*.

### IFN-γ Release Assay (IGRA)

Antigens were diluted in RPMI 1640 to the appropriate concentrations and 25 μL of the antigen suspension was dispensed in duplicate in 96-well round bottom cell culture plates (Corning, Amsterdam, The Netherlands). Pokeweed Mitogen (PWM, 5 μg/mL, Prionics™, Thermo Fisher Scientific, Schlieren-Zürich, Switzerland) served as positive control, pure medium as negative control. Two hundred fifty microliter of heparinized blood was added per well (for final antigen concentrations see Table 1). The plates were incubated for 20 h at 37 ± 2°C, 5% CO_2._ After centrifugation at 300 × g for 10 min, the supernatants were collected and stored at −18 ± 3°C until analysis. IFN-γ was measured with two different ELISA systems depending on the purpose of the analysis. For follow-up of the PPDj-induced IFN-γ response during the course of the animal experiment, an in-house capture ELISA using monoclonal antibodies against bovine IFN-γ was performed essentially as described elsewhere (28). The IFN-γ levels induced by the antigens to be tested were analyzed with the BOVIGAM® test system (Prionics™, Thermo Fisher Scientific, Schlieren-Zürich, Switzerland) following the instructions of the manufacturer. Samples were tested in duplicate and the mean OD values calculated for further data processing.

### Serum Preparation and Antibody Detection

Blood without anti-coagulants was kept for 2–3 h at RT, centrifuged at 2,000 × g for 20 min, the serum recovered, aliquoted and stored at −20°C until use. Antibodies against mycobacterial antigens were detected with a modified protocol of the ID Screen Mycobacterium Avium Indirect ELISA (ID Vet SARL, Montpellier, France). Instead of the multi-species peroxidase conjugate provided with the kit, horseradish peroxidase labeled anti-bovine immunoglobulin (Prionics™, Thermo Fisher Scientific, Schlieren-Zürich, Switzerland) was applied as conjugate. The antibody response is demonstrated by the sample-to-positive ratio (S/P) as recommended by the manufacturer.

### Fecal Culture

Fecal culture was performed as described previously (28) with minor modifications. In brief, three grams of feces were decontaminated for 48 h in 30 mL of 0.75% HPC in upright position, enabling sedimentation of bacteria. Supernatants were discarded and 200 μL of the pellet were transferred on each of four slopes of Herrold's Egg Yolk Medium with Mycobactin J and Amphotericin, Nalidixic acid and Vancomycin (ANV, HEYM, Becton Dickinson, Heidelberg, Germany). Cultures were incubated at 37 ± 2°C. The slopes were checked every 2 weeks for contamination and occurrence of visible colonies. As soon as colonies became visible, MAP, and MAH were confirmed by PCR as described above and the samples were classified positive. Incubation of negative culture slopes from MAP-inoculated goats was stopped after 6 months and of samples from MAH inoculated goats after 8 weeks. The results were recorded as positive or negative bacterial growth.

### Necropsy, Collection of Tissue Samples, Histology, and Immunohistochemistry

At necropsy, gross lesions were documented and tissues sampled for histology, immunohistochemistry (IHC) and cultivation of mycobacteria. Representative samples were collected from intestine (duodenum, 4 sites of jejunum, cecum, and colon), gut-associated lymphoid tissues (GALT; 2 jejunal Peyer's patches (JPPs), 2 sites of ileal Peyer's patch (IPP), GALT next to the ileocaecal valve and in the proximal colon) and regional intestinal lymph nodes. For histology, paraffin sections were prepared and stained with haematoxylin and eosin (HE). Mycobacteria and mycobacterial antigen were labeled by IHC in all sites of intestine, GALT and regional intestinal lymph nodes. The method has been described for MAP and MAH in detail (27, 39). Briefly, sections were pre-treated with trypsin (0.1%, 37°C, 20 min) for antigen retrieval. A polyclonal rabbit anti-MAP serum (Dako, Glostrup, Denmark) was used as primary, peroxidase-conjugated goat anti-rabbit IgG (Dianova, Hamburg, Germany) as secondary antibody and 3-amino-9-ethyl carbazol as chromogen. Sections were counterstained with hematoxylin.

### Tissue Culture

Tissues were processed as described elsewhere (28) and decontaminated with 0.9% HPC for 24 h at room temperature. After centrifugation, supernatants were discarded and the pellets re-suspended with 1 mL of sterile phosphate buffered saline. Two hundred microliter of the re-suspended pellet were transferred to each of four slopes of HEYM. The cultures were incubated and bacterial growth recorded as described above.

### Data Analysis and Statistics

#### In-house Capture ELISA for Follow-Up of Johnin-Induced IFN-γ Response

Corrected OD values (cOD) were calculated by correcting the mean OD values of the samples from stimulation assays tested on one ELISA plate for the mean OD of the negative ELISA control. IFN-γ responses were expressed by the sample to positive ratio (S/P), which was calculated by dividing the cOD of each sample by the cOD of the positive ELISA control.

#### BOVIGAM Test System for Antigen Testing

First, cOD values were calculated as described above. For all antigens that were tested in two concentrations, it was necessary to determine which concentration to use in further data analysis. Therefore, the linear correlation (Pearson's correlation coefficient *r*) between the cOD values of both antigen concentrations was calculated. If *r* was higher than 0.5 and the statistical significance level *P* < 0.0001, only one concentration of antigen (10 μg/mL) was selected (Supplementary Table 1). IGRA response is presented as the difference between the cOD of antigen stimulated samples and the cOD of the unstimulated sample of the same animal on the same day [S-N].

#### Statistical Tests

Group differences per time point were tested with One-way ANOVA using the Tukey-Kramer *post-hoc* test for all pairwise comparisons. Spearman rank correlation coefficients were calculated to establish the relation between the IFN-γ response after stimulation with PPDj and after stimulation with selected protein antigens. Data analysis was performed using IBM SPSS Statistics Version 19 (19.0.0.2) and MedCalc Statistical Software version 13.1.0 (MedCalc Software bvba, Ostend, Belgium; http://www.medcalc.org; 2014).

## Results

### Confirmation of Mycobacteria Infection in Goats Contributing to Antigen Testing

All inoculated goats allocated to the sub-groups for antigen testing fulfilled several criteria indicating mycobacterial infection. Increased body temperature (39.6°C up to 40.9°C) and mild depression were noted in the MAH inoculated goats from the end of the inoculation period until 7–10 wpi. Subsequent to that, these animals showed no clinical signs. During the whole course of the study, the MAP inoculated goats did not develop clinical signs indicative for MAP infection (data not shown). Shedding of the inoculum strains varied between the MAP and the MAH groups. MAH was re-isolated from feces of all goats 1 day after oral inoculation (dpi) and of 3/8 goats 3–6 wpi. No further shedding was detected until the end of the experiment, despite the more than 10-fold higher inoculum compared to MAP. MAP was re-isolated from feces of 2/8 goats at 1 dpi and from all goats 3–6 and 7–10 wpi. Afterwards, fecal shedding of MAP was observed regularly in 3 goats and intermittently in the other 5 goats until the end of the experiment (for semi-quantitative data see Supplementary Table 2). Infection was confirmed at the end of the trial by re-isolation of the bacterial strain used for inoculation from tissues of the gastrointestinal tract (GIT) and/or by lesions characteristic for mycobacterial infections. Re-isolation of bacteria from the GIT was not possible from three of the MAH inoculated goats, but characteristic gross and histopathologic lesions were present and mycobacteria were demonstrated in the lesions by IHC, except for two MAH and two MAP-inoculated animals (Table 2). Indications of MAP or MAH infection were not found in any of the goats of the control group during the course of the study.

**Table 2:** Results of fecal and tissue culture of MAP/MAH, presence of lesions and detection of mycobacteria by IHC.

**Group**	**Animal No**	**Fecal shedding**	**Re-isolation from GIT**	**Intestine and/or GALT**		**Regional intestinal lymph nodes**	
				**Lesions**	**IHC**	**Lesions**	**IHC**
Control	0045	–	–	–	–	–	–
	0046	–	–	–	–	–	–
	0047	–	–	–	–	–	–
	0049	–	–	–	–	–	–
	0050	–	–	–	–	–	–
	0051	–	–	–	–	–	–
	0052	–	–	–	–	–	–
	0054	–	–	–	–	–	–
MAH	0024	+	–	+	–	+	+
	0027	+	+	+	+	+	+
	0029	+	+	+	+	+	+
	0033	+	+	+	+	+	+
	0034	+	+	+	+	+	+
	0036	+	–	+	+	+	+
	0039	+	–	+	+	+	+
	0041	+	+	+	+	+	+
MAP	0005	+	+	+	+	+	+
	0007	+	+	+	+	–	–
	0011	+	+	+	+	+	+
	0012	+	+	+	+	+	+
	0016	+	+	+	+	+	+
	0020	+	+	+	+	+	+
	0021	+	+	+	+	+	+
	0023	+	+	+	–	+	+

–*negative/not present/not detected; + positive/present/detected*.

**Figure 2:**
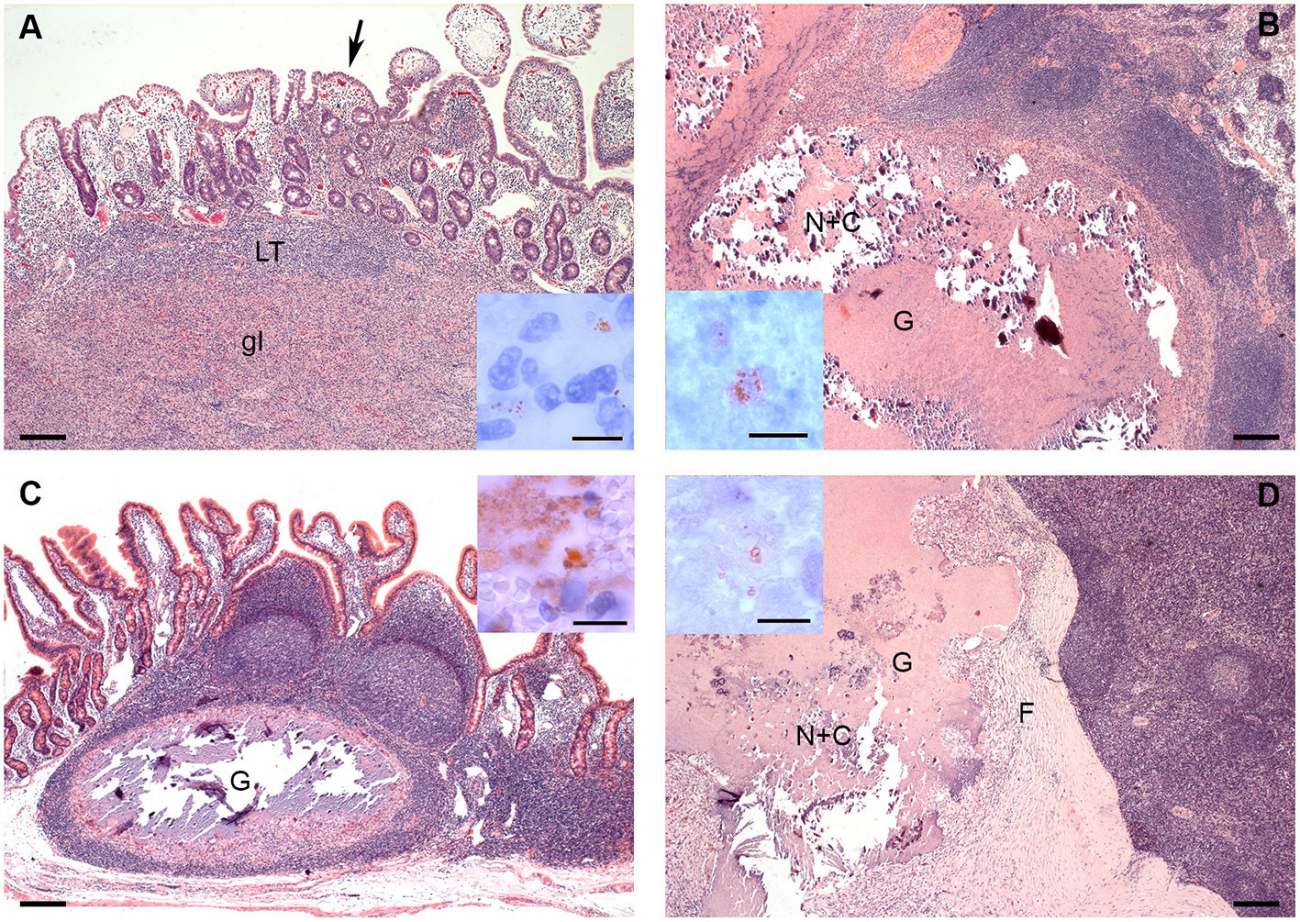
**(A–D)**. Histologic Lesions in goats inoculated with MAP **(A,B)** and MAH **(C,D)**. **(A)** The granulomatous infiltrate (gI) replaces most of the lymphoid tissue (LT) of a JPP. There are multifocal granulomatous infiltrates (arrow) in the overlying lamina propria. HE-stain; **Inset**: A few mycobacteria are present in the granulomatous infiltrate in the lamina propria. IHC anti-MAP; **(B)** Part of a large multicentric granuloma (G) with extensive central necrosis and calcification (N+C) in an ileocolic lymph node. HE-stain; **Inset**: A few mycobacteria are present within the necrosis. IHC anti-MAP; **(C)** Well demarcated granuloma with central necrosis and calcification in a JPP. There are no lesions in the adjacent lymphoid tissue or mucosa. HE-stain; **Inset**: There is a focal area with many mycobacteria at the edge of the necrosis. IHC anti-MAP; **(D)** Part of a large multicentric granuloma (G) with extensive central necrosis and calcification (N+C) demarcated by a thick fibrous capsule (F) in an ileocolic lymph node. HE-stain; **Inset**: A few mycobacteria are present within the necrosis. IHC anti-MAP. Bars = 200 μm, inset bars = 10 μm.

Differences in distribution and characteristics of lesions were seen in goats infected with MAP compared to those infected with MAH (Figures 2A–D). All goats inoculated with MAP had focal to multifocal granulomatous infiltrates in all parts of GALT. Lesions were detected most consistently in JPP (8/8 goats, Figure 2A) and least often in IPP (3/8 goats). Granulomatous infiltrates were also distributed throughout the small intestine in 6 of the 8 goats. A few mycobacteria were found in about 50% of the lesions (Figure 2A inset). Granulomas and granulomatous infiltrates were present in regional intestinal lymph nodes (ILN) of 7 of the 8 goats, most often affecting the ileocolic lymph nodes. Most granulomas in the lymph nodes were large with multi-centric areas of necrosis and calcification (Figure 2B). A few mycobacteria were found in those granulomas (Figure 2B inset).

In goats inoculated with MAH, lesions were limited to JPP and IPP and did not affect other parts of the intestine. They were more frequent in IPP (7/8 goats) than in JPP (5/8 goats). Lesions consisted of solid infiltrates of epitheloid cells with minimal necrosis or small granulomas with monocentric necrosis and calcification (Figure 2C). A few mycobacteria were detected in most of the lesions (Figure 2C inset). Regional ILN were more extensively and severely affected than intestines. Granulomas and less frequently granulomatous infiltrates were detected in regional ILN of the small intestine of all goats (8/8 goats) and colonic lymph nodes of 3 out of 8 goats. Small monocentric and large multicentric granulomas predominated (Figure 2D). In 3 out of 8 goats, granulomas had a marked fibrotic organization. A few mycobacteria were seen in all lesions (Figure 2D inset).

No lesions related to mycobacterial infections were present in any of the mock-inoculated control goats (Table 2).

**Figure 3:**
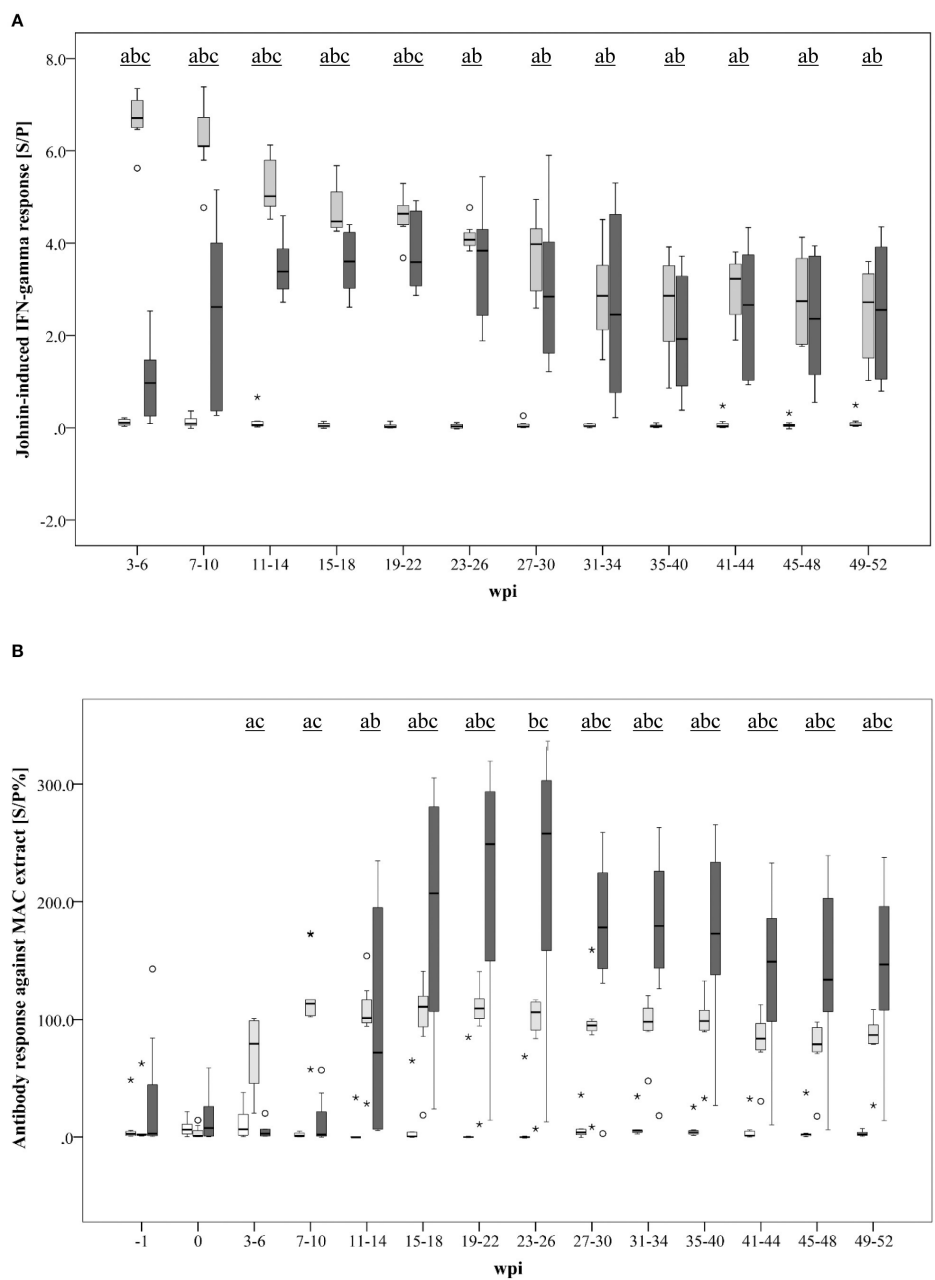
**(A, B)** Kinetics of the specific immune response of MAH and MAP-inoculated goats vs. non-infected control goats. **(A)** PPDj-induced whole blood IFN- response [S/P]; **(B)** serum antibody response against a MAC extract containing common antigens of MAH and MAP measured by ELISA [S/P%]. Box and Whisker Plot represents median value, 25% and 75% percentiles (box), range, outlier values (■), and extreme values (*). Groups: control, MAH, MAP. Different letters indicate significant differences between the groups: a - control vs MAH, b - control vs MAP, c - MAH vs MAP (Tukey-Kramer *post hoc* test, *P* < 0.05).

A marked Johnin-induced whole blood IFN-γ response was detected in all inoculated goats, however, with different kinetics in MAH and MAP-infected animals (Figure 3A). While the MAH infected group showed maximum IFN-γ levels already 3–6 wpi with gradual decrease until the end of the study at 52 wpi, the response of the group infected with MAP increased steadily, peaked only 23–26 wpi, and decreased slowly afterwards. In general, inter-individual variation of the responses was higher in the MAP-inoculated animals.

Antibody formation against antigens that are shared by MAH and MAP (present in the *Mycobacterium avium* Complex extract used in the assay) was detected 3–6 wpi in MAH-inoculated goats but was delayed in MAP-inoculated animals (11–14 wpi) (Figure 3B). Antibody levels of the MAH group remained roughly at the same level from 7 to 10 wpi until the end of the experiment. A high inter-individual variation was noted in the MAP group. Median antibody levels of the MAP group exceeded those of the MAH group from 15 to 18 wpi onwards.

### IFN-γ Response to Mycobacterial Antigens at Selected Time Points During Infection

**Figure 4:**
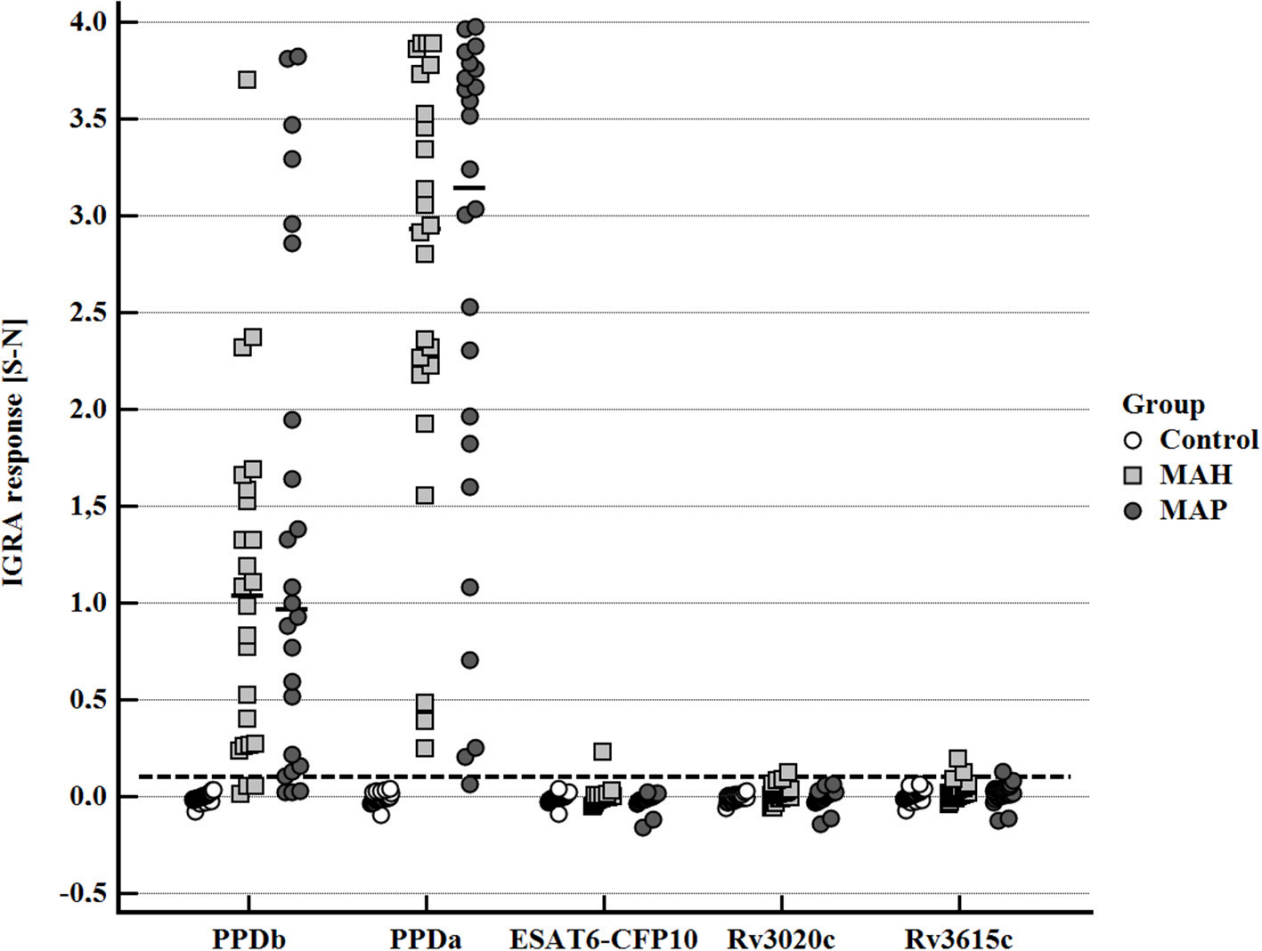
Whole blood IFN-γ response of MAH or MAP inoculated goats vs. control animals induced by bovine PPD (PPDb, 300 U), avian PPD (PPDa, 250 U), and recombinant *M. bovis* antigens ESAT6-CFP10, RV3020c, and RV3615c (5 μg/mL each). Data of three time points (19–22, 27–30, and 45–48 wpi) are summarized. Each dot represents one animal; bars represent medians. The dashed line indicates the lower limit of a positive IFN-γ response [S- N] to stimulation with either PPDb or PPDa.

After stimulation with the standard antigens applied for the diagnosis of bovine tuberculosis by IGRA, PPDb, and PPDa, a distinctive IFN-γ response (S-N ≥0.1) was observed in whole blood supernatants of the majority of MAH and MAP-inoculated goats, while the control animals had a minimal response (Figure 4). However, the PPDa response was considerably higher than the PPDb response except for one MAH-inoculated goat. Thus, by applying the standard interpretation criteria for test positivity of the BOVIGAM IGRA (cOD PPDb—cOD PPDa ≥ 0.1, Supplementary Figure 1) these animals tested negative for bTb. In contrast, stimulation of whole blood with the *M. bovis* antigens ESAT6-CFP10, Rv3020c, and Rv3615c elicited no or only very weak IFN-γ production (Figure 4). Applying a cut-off of S-N ≥0.1 one MAH inoculated goat each after stimulation with ESAT6-CFP10 or Rv3020c presented as test positive. Further two MAH-inoculated and one MAP-inoculated goats showed S-N values ≥ 0.1 after stimulation with Rv3615c.

**Figure 5:**
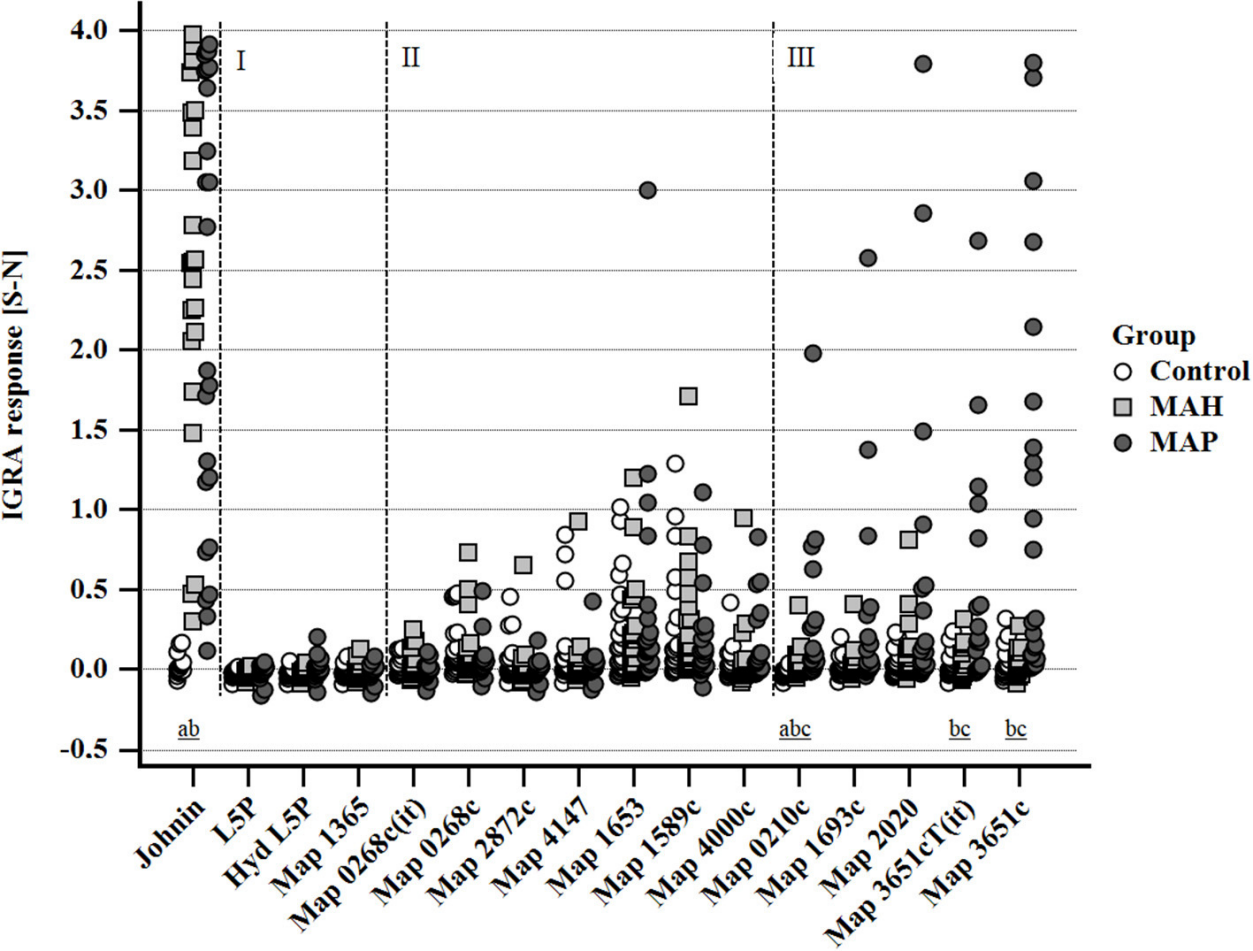
Whole blood IFN-γ response of MAH or MAP inoculated goats vs. control animals induced by PPDj (5 μg/mL) and different MAP antigens (10 μg/mL each). Cluster I: very weak IFN-γ responses in all three groups of animals; cluster II: IFN-γ responses with similar magnitude and no group differences; cluster III: increased IFN-γ responses in MAP-inoculated goats. Data of three time points (19–22, 27–30, and 45–48 wpi) are summarized. Each dot represents one animal. Different letters indicate significant differences between the groups: a—control vs. MAH, b—control vs. MAP, c—MAH vs. MAP (Tukey-Kramer *post-hoc* test, *P* < 0.05).

Stimulation with PPDj (5 μg/ml) induced a marked IFN-γ response of whole blood from MAH and MAP-inoculated goats, although with large inter-individual variation. The potential of MAP lipid and protein antigens to trigger IFN-γ production varied (Figure 5). Very weak IFN-γ responses were induced by L5P, Hyd L5P, and Map 1365, respectively, with no group differences. Several antigens (Map 0268cT(it), Map 0268c, Map 2872c, Map 4147, Map 1653, Map 1589c, and Map 4000c) elicited similar, low levels of IFN-γ production in blood cells of both controls and mycobacteria-inoculated animals. However, antigens Map 0210c, Map 1693c, Map 2020, Map 3651cT(it), and Map 3651c stimulated an increased IFN-γ response of blood cells of several MAP-inoculated goats which was not observed in any MAH-inoculated or control animals (Figure 5). The number of positive responders differed between the antigens, with Map 3651c being the most prolific stimulant. Inter-individual and temporal variation of the responses was considerable (Supplementary Figure 2). The most pronounced antigen-induced IFN-γ responses and the highest numbers of responders were observed 19–22 and 27–30 wpi. Two of the eight MAP-inoculated animals responded to the five antigens consistently at both time points. The same animals responded to Map 3651cT(it) and Map 3651c in most cases, although the IFN-γ levels were higher after stimulation with Map 3651c. At 45–48 wpi, the antigen-induced IFN-γ levels of the MAP group were much less pronounced (Supplementary Figure 2).

**Figure 6:**
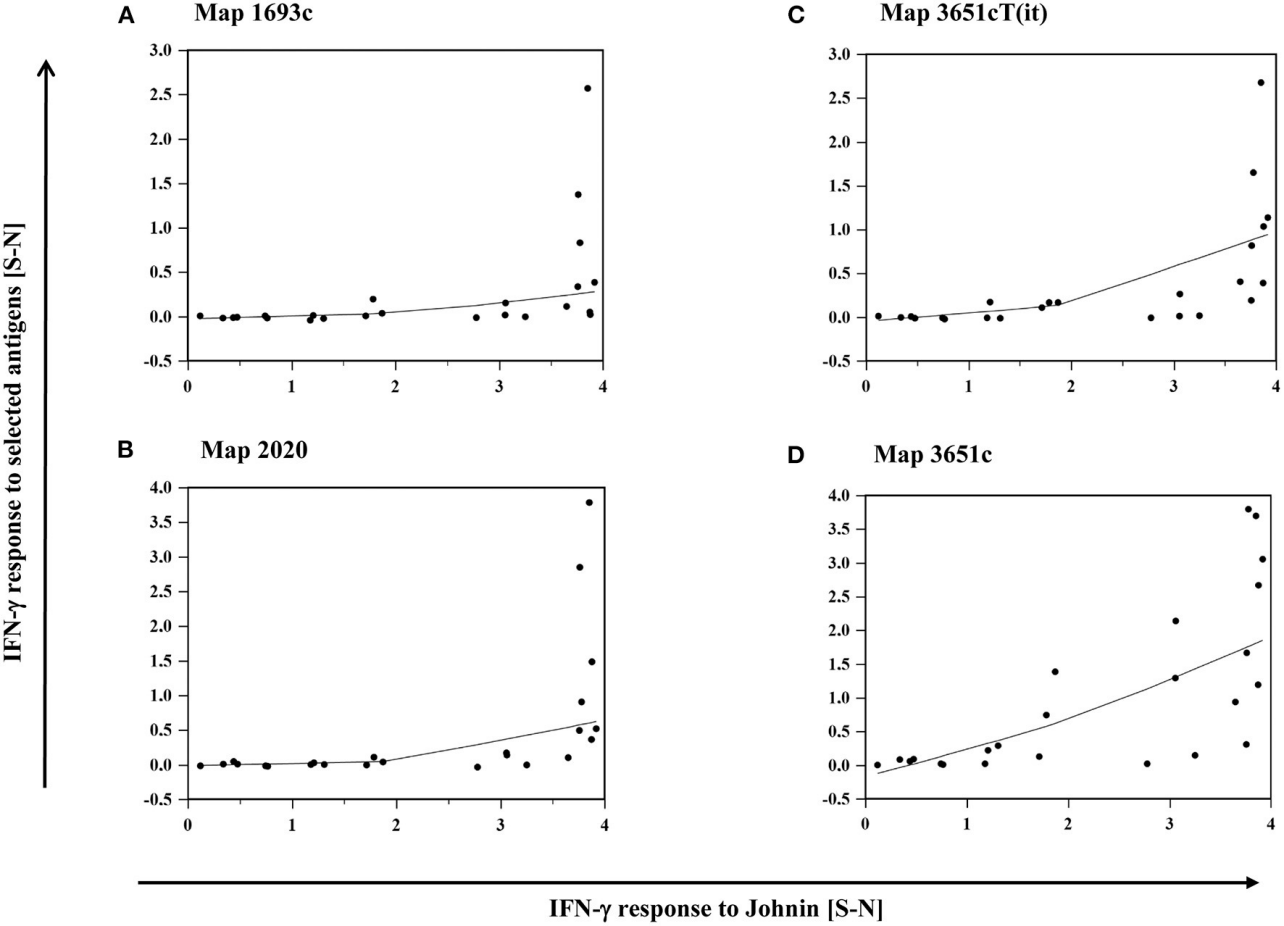
**(A–D)**: Relation between the IFN-γ response of MAP inoculated goats to PPDj and selected MAP antigens, **(A)** Map 1693, **(B)** Map 2020, **(C)** Map 3651cT(it), and **(D)** Map 3651c. Data of three time points (19–22, 27–30, and 45–48 wpi) are summarized. Each dot represents one animal. Line: local regression smoothing trend line with smoothing span of 80%.

The intensity of IFN-γ production, represented by S-N values, after stimulation with each of the antigens mentioned above correlated with their response to PPDj (Supplementary Table 3). Antigen-induced responses were predominantly observed in animals that mounted high IFN-γ responses to PPDj (Figures 6A–D).

## Discussion

Only limited data are available regarding the diagnostic performance of the IGRA using specific protein and lipid antigens of MAP in goats. Recently, 16 MAP recombinant proteins and 5 lipids were tested in a field trial in goats from herds with or without history of paraTb (21). Ten of the recombinant proteins were selected as potential candidates for the detection of MAP infection in young goats. However, these antigens elicited positive IGRA responses in a considerable proportion of 1-year old goats from historically paraTb non-suspect herds, raising uncertainties about the specificity of the antigens and the true infection status of the goats.

The present study included *post mortem* confirmation of the infection status of the inoculated animals and the controls at the end of the study by culture of MAP or MAH from tissues or by the occurrence of gross or histopathologic tissue lesions. Infection was confirmed in all MAP and MAH inoculated goats, and the control goats showed no signs of infection. Furthermore, animals were followed closely during the course of the experiment. The time points of antigen testing (19–22, 27–30, 45–48 wpi) were pre-determined based on the course of the specific whole blood IFN-γ response during a preceding goat study (28). At these time points, all animals of the MAP and MAH groups mounted a clear PPDj-induced whole blood IFN-γ response that differed significantly from the control animals, despite large individual variation. In addition, antibodies against common mycobacterial antigens were also detectable.

The MAP-derived lipopentapeptide L5P was a promising antigen candidate for antibody detection and IGRA in MAP-infected cattle (11, 19). However, only very weak IFN-γ responses and no differences between the three treatment groups were observed in the goats after whole-blood stimulation with chemically synthesized L5P and Hyd L5P. A similar lipopeptide preparation, Para-LP-01 isolated from C-type strain K-10 (40), failed to induce detectable IFN-γ responses in S-type-exposed sheep (20). Since S-type MAP strains do not produce L5P but rather a shorter lipopeptide (41), this might explain the absence of a response in those sheep. Other reasons discussed for a missing response are more likely to apply for L5P and its hydro-soluble form. It might be possible that a whole blood assay is not suited to detecting an IFN-γ response to a lipopeptide, because activation of lymphocytes by lipids is different to protein antigens due to different antigen presentation pathways and may require higher antigen concentrations and close contact with purified cell subsets (20). However, the antigen concentrations applied in our study (10 and 20 μg/mL) were much higher than those reported by others, which ranged from 0.5 to 2 μg/mL in the sheep study (20) and were 5 μg/mL in the cattle study (11). Instead, immunosuppressive and even cytotoxic effects of the lipopentapeptide, which have been discussed previously (20), cannot be excluded. It is also conceivable, but was not studied so far, that the *in vivo* configuration of the epitope on the surface of the mycobacterial envelope, which is limited to 5 amino acids, differs from the synthetic compound used in the *in vitro* assay. Furthermore, species differences in the expression of CD1 molecules, which are engaged in the antigen presentation of lipids and lipopeptides (42), have also to be taken into account, because MAP-exposed cattle are able to mount an IFN-γ response to L5P, while sheep and goats are not.

Depending on their IFN-γ responses, MAP-derived protein antigens were divided into two categories: (a) non-discriminatory antigens, which induced similar levels of IFN-γ production by blood cells of goats in all three treatment groups; and, (b) antigens that stimulated an increased IFN-γ response in MAP-exposed goats.

The protein antigen Map 1365, which had been shown to induce a marked IFN-γ response in 5/7 sub-clinically infected sheep (15), elicited only very low IFN-γ levels and was not discriminatory in goats. Based on results from sheep and cattle, Map 0268c was suggested to have the ability to discriminate groups of infected and uninfected animals (15, 16). Two preparations of this antigen were tested here and, although one of the preparations stimulated increased IFN-γ release of blood cells of individual goats, they did not enable discrimination between the treatment groups. The same was true for Map 2872c, Map 4147, Map 1653, and Map 1589c, which induced similar responses of individual goats of all three groups. It cannot be ruled out that the non-specific responses of control and MAH-infected goats were age dependent, because the animals were younger than 12 months when they were tested. In another goat study, decreased specificity of the IGRA response was reported for Map 1653 and Map 1589c in 6-month old goats, while specificity was increased in 20-month-old goats. Map 2872c as well as Map 4147 were not analyzed (21). Non-specific responses to individual mycobacterial protein antigens have been reported in young animals by others (43). They were explained by NK-cell mediated IFN-γ production (44).

Map 4000c elicited increased IFN-γ levels in one MAH and three MAP-infected goats compared to controls. This antigen seems not to be MAP-specific, because the magnitude of the IFN-γ response of MAH and MAP-infected goats was similar. However, in the study mentioned above (21), Map 4000c showed higher specificity in 6-month old and 20-month old goats than other proteins.

The protein antigens Map 0210c, Map 1693c, Map 2020, Map 3651cT(it), and Map 3651c elicited IFN-γ production in individual MAP-infected goats that was considerably higher than responses of control and MAH infected animals. Our findings are in accordance with other studies, since Map 0210c, Map 2020, and Map 3651c belonged to a set of MAP antigens that was proposed to optimize the sensitivity of a caprine IGRA. However, Map 1693c was considered to be of little interest (21). Map 3651c is the antigen with the most universal diagnostic potential. It was shown to have the ability to discriminate groups of infected and uninfected sheep (15) and to elicit increased IFN-γ levels in cattle from paraTb high-risk farms (16). Two different recombinant antigen preparations of Map 3651c were included in the present study, a full lengths antigen, Map 3651c (21, 36) and a truncated protein, Map 3651cT(it) (16). Obviously, the capacity of the truncated protein to induce IFN-γ responses was reduced compared to the full lengths antigen, which may be due to altered epitope configuration.

Despite large individual variation, increased IFN-γ responses to individual protein antigens were associated with high PPDj-induced IFN-γ levels. Like PPDb and PPDa, PPDj is a complex antigen mixture that elicits IFN-γ production by a multitude of T lymphocytes that respond to different individual antigens. However, the proportion of T cells in blood with the ability to respond to this mixture is very small. In the majority of samples, the proportion of IFN-γ^+^ T cells expressing either CD4 or CD8 was below 2% after stimulation of caprine blood with PPDj (45). It has to be assumed that these cells represent recirculating T cells that continuously exit into efferent lymphatics to return to the blood circulation from lymph nodes draining the local infection sites (46). The proportion of cells that respond to one individual antigen is only a small fraction of all responding cells. Obviously, only when sufficient numbers of lymphocytes responding to PPDj are present in blood, the number of cells responding to one individual immunogenic antigen is high enough to induce an IFN-γ response that can be detected by ELISA. Likewise, it has to be considered, that low amounts of cytokine are scavenged by receptors of other cells in culture, preventing the accumulation of amounts of IFN-γ that are detectable (47). Therefore, it seems necessary to use mixtures of specific protein or peptide antigens for whole blood stimulation to elicit sufficiently high IFN-γ levels.

PPDb and PPDa induced a marked whole blood IFN-γ response of the MAH and MAP-inoculated goats, while the controls did not respond. This was not un-expected, because these complex antigen mixtures contain several antigens common to different mycobacteria species (9). However, IFN-γ levels in the PPDa-stimulated samples of both groups were much higher than in the PPDb-stimulated samples pointing to the existence of *M. avium*-complex specific antigens within PPDa. When the analysis algorithm of the BOVIGAM Test System for bTb was applied (negative: cOD PPDb—cOD PPDa < 0.1), all but one sample of the MAH and MAP-inoculated goats resulted in values <0.1, confirming that the animals were bTb free. This underlines the necessity of including PPDa in the test system when tuberculins serve as test antigens for bTb diagnosis to compensate for non-specific stimulation, especially in regions where paraTb is endemic.

As expected, the *M. bovis* antigens ESAT6-CFP10, Rv3020c, and Rv3615c induced only low levels of IFN-γ production in MAH or MAP-inoculated goats highlighting their specificity to be recognized primarily in animals or humans infected with Mtb complex pathogens such as *M. bovis* or *M. tuberculosis*. Utilization of these antigens within the IGRA for the diagnosis of bTb may be another approach to increase the specificity of the test in paraTb-infected herds. However, diagnostic interpretation criteria for these antigens have to be determined in naturally MAP-infected animals including officially tuberculosis free goat herds. A similar strategy was followed in a recent study in cattle suggesting that interference caused by paraTb vaccination could be overcome by the application of ESAT6-CFP10 and Rv3615c as IGRA antigens (48). Peptide cocktails derived from ESAT-6 and CFP-10 discriminated also between *M. bovis* infection and vaccination, when vaccine candidates for bTb were tested in cattle (49).

## Conclusions

Four MAP-derived protein antigens proved to be potential candidates to increase the specificity of the whole-blood IGRA for the diagnosis of MAP infection in young goats. However, since positive antigen-derived IFN-γ levels were only observed in samples with high IFN-γ responses to PPDj, further work is required to find out if test sensitivity will be increased to a sufficient degree when antigen mixtures are applied.

*M. bovis* antigens ESAT6-CFP10, Rv3020c, and Rv3615c do not elicit IFN-γ responses in MAP and MAH infected goats confirming the specificity of these IGRA reagents for the diagnosis of bTb in paraTB-infected goat herds.

## Data Availability Statement

The original contributions presented in the study are included in the article/Supplementary Material, further inquiries can be directed to the corresponding author/s.

## Ethics Statement

The animal study was reviewed by the Committee on the Ethics of Animal Experiments of the State of Thuringia, Germany, and approved by the competent authority, the Thuringian State Office for Consumer Protection (Permit Number: 22-2684-04-04-002/12, date of permission 12.12.2012).

## Author Contributions

HV coordinated the transnational research project. HK and EL-T conceived the study, planned and supervised the animal experiment, and drafted the manuscript. EL-T performed necropsies and macroscopic and histologic assessment of lesions. HK supervised bacterial culture and immunological experiments and analyzed the data. KS, VH, DB, PW, FB, SB, CG, and HV prepared and provided antigens. All authors discussed the results, commented on the manuscript, and read and approved the final manuscript.

## Conflict of Interest

HV is co-inventor on patents protecting the application of Rv3615c for the diagnosis of tuberculosis. FB and SB are co-inventors on patents WO 2009/053844 A1 (2008) and WO 2018/115183 (2017). The remaining authors declare that the research was conducted in the absence of any commercial or financial relationships that could be construed as a potential conflict of interest.

## Footnotes

^1^http://www.fao.org/faostat/en/#data/TA
