# Supplementary material for: Interferon-γ Response of Mycobacterium avium subsp. paratuberculosis Infected Goats to Recombinant and Synthetic Mycobacterial Antigens
Source: Front Vet Sci. 2021 Mar 26;8:645251. doi: 10.3389/fvets.2021.645251 (PMC8034290; doi:10.3389/fvets.2021.645251)
Supplement: Supplementary file 1 [file Data_Sheet_1.pdf]

SUPPLEMENTAL MATERIAL

TABLES

Table S1: Linear correlation between IGRA OD values after stimulation with antigens in two different concentrations (10 vs. 20 µg/mL)

Antigen	n	Correlation coefficient r*	Significance level *P*<	LL CI for r	UL CI for r	Concentration selected (µg/mL)
**L5P**	72	0.5331	0.0001	0.3439	0.6807	10
**Hyd L5P**	72	0.8227	0.0001	0.7302	0.8856	10
**Map 0268c**	72	0.9003	0.0001	0.8449	0.9366	10
**Map 0268c(it)**	72	0.8149	0.0001	0.7189	0.8804	10
**Map 1365**	72	0.7633	0.0001	0.6458	0.8455	10
**Map 3651cT(it)**	72	0.9952	0.0001	0.9923	0.997	10
**Map 4147**	30	0.9527	0.0001	0.9021	0.9775	10
**Map 2872c**	30	0.9771	0.0001	0.9519	0.9892	10
**Map 1589c**	72	0.981	0.0001	0.9698	0.9881	10
**Map 1653**	72	0.959	0.0001	0.935	0.9742	10
**Map 3651c**	72	0.9949	0.0001	0.9919	0.9968	10
**Map 1693c**	72	0.9874	0.0001	0.9798	0.9921	10
**Map 0210c**	72	0.9962	0.0001	0.9939	0.9976	10
**Map 4000c**	72	0.839	0.0001	0.7538	0.8964	10
**Map 2020**	72	0.9786	0.0001	0.9659	0.9866	10

*Pearson`s correlation coefficient; LL CI lower limit of the confidence interval; UL CI upper limit of the confidence interval.

Table S2: Intensity of faecal shedding of MAH and MAP in the course of the study.

Group	Animal No.	ai	1 dpi	3-6 wpi	7-10 wpi	11-14 wpi	15-18 wpi	19-22 wpi	23-26 wpi	27-30 wpi	31-34 wpi	35-40 wpi	41-44 wpi	45-48 wpi
	**0045**	0	0	0	0	0	0	0	0	0	0	0	0	0
	**0046**	0	0	0	0	0	0	0	0	0	0	0	0	0
	**0047**	0	0	0	0	0	0	0	0	0	0	0	0	0
**Control**	**0049**	0	0	0	0	0	0	0	0	0	0	0	0	0
	**0050**	0	0	0	0	0	0	0	0	0	0	0	0	0
	**0051**	0	0	0	0	0	0	0	0	0	0	0	0	0
	**0052**	0	0	0	0	0	0	0	0	0	0	0	0	0
	**0054**	n.a.	0	0	0	0	0	0	0	0	0	0	0	0
	**0024**	0	+[§]	0	0	0	0	0	0	0	0	0	0	0
	**0027**	0	+	c	0	0	0	0	0	0	0	0	0	0
	**0029**	n.a.	+	+	0	0	0	0	0	0	0	0	0	0
**MAH**	**0033**	0	+	0	0	0	0	0	0	0	0	0	0	0
	**0034**	0	+	+	0	0	0	0	0	0	0	0	0	0
	**0036**	0	++	++	0	0	0	0	0	0	0	0	0	0
	**0039**	0	+++++	0	0	0	0	0	0	0	0	0	0	0
	**0041**	0	+	0	0	0	0	0	0	0	0	0	0	0
	**0005**	0	0	++	+	0	+	+	+	+	+	+	++	+
	**0007**	0	0	++	+	0	0	0	0	0	0	0	0	0
	**0011**	0	0	+	+	++++	++++	+	+	++	++	+++++	+++++	++++
**MAP**	**0012**	0	0	++	++	+++	+++	++	++	++	++	++	+	+
	**0016**	0	0	++	+	+	0	+	0	+	0	0	0	0
	**0020**	0	+	++	+	+++	+++++	+++	+	0	0	+	0	0
	**0021**	0	+	+	+++	++++	++	+	+	+	+	+	0	0
	**0023**	0	0	+	+	+	++	++++	+	+	0	0	0	0

ai before inoculation; n.a. sample not available; c culture contaminated;

[§]colony growth score: 0 no growth, + GI ≤ 15.0, ++ GI > 15.0 ≤ 30.0, +++ GI > 30.0 ≤ 45.0, ++++ GI > 45.0 ≤ 60.0, +++++ GI > 60.0.

Colony growth was scored using the growth indices (GI) calculated as described by Köhler et al. (28). In brief, colony counts were estimated semi-quantitatively by a colony score (CS) from 1 to 5, with 1 = 0 – 10; 2 = 11 – 20; 3 = 21 – 50; 4 = 51 – 100 individual colonies per slope; 5 = bacterial lawn, and the week of appearance (WA) was recorded. To correct for the time until colonies became visible, a growth index (GI) allowing a numerical estimation of the bacterial concentration in the samples was calculated by the formula: GI = CS × 100/WA. The mean GI of the four corresponding slopes was determined.

Table S3: Rank correlation between IFN-γ responses after re-stimulation with Johnin and selected Map antigens

**Antigen**		**Map 0210c**		**Map 1693c**		**Map 2020**		**Map 3651c**		**Map 3651cT(it)**	
	**n**	**ρ***	***P*<**	**ρ**	***P*<**	**ρ**	***P*<**	**ρ**	***P*<**	**ρ**	***P*<**
**Johnin**	24	0.768	0.0001	0.746	0.0001	0.743	0.0001	0.831	0.0001	0.814	0.0001
**Map 0210c**	24	-	-	0.795	0.0001	0.657	0.0005	0.657	0.0005	0.853	0.0001
**Map 1693c**	24	0.795	0.0001	-	-	0.807	0.0001	0.806	0.0001	0.900	0.0001
**Map 2020**	24	0.657	0.0005	0.807	0.0001	-	-	0.885	0.0001	0.845	0.0001
**Map 3651c**	24	0.657	0.0005	0.806	0.0001	0.885	0.0001	-	-	0.851	0.0001

*Spearman rank correlation coefficient

FIGURES

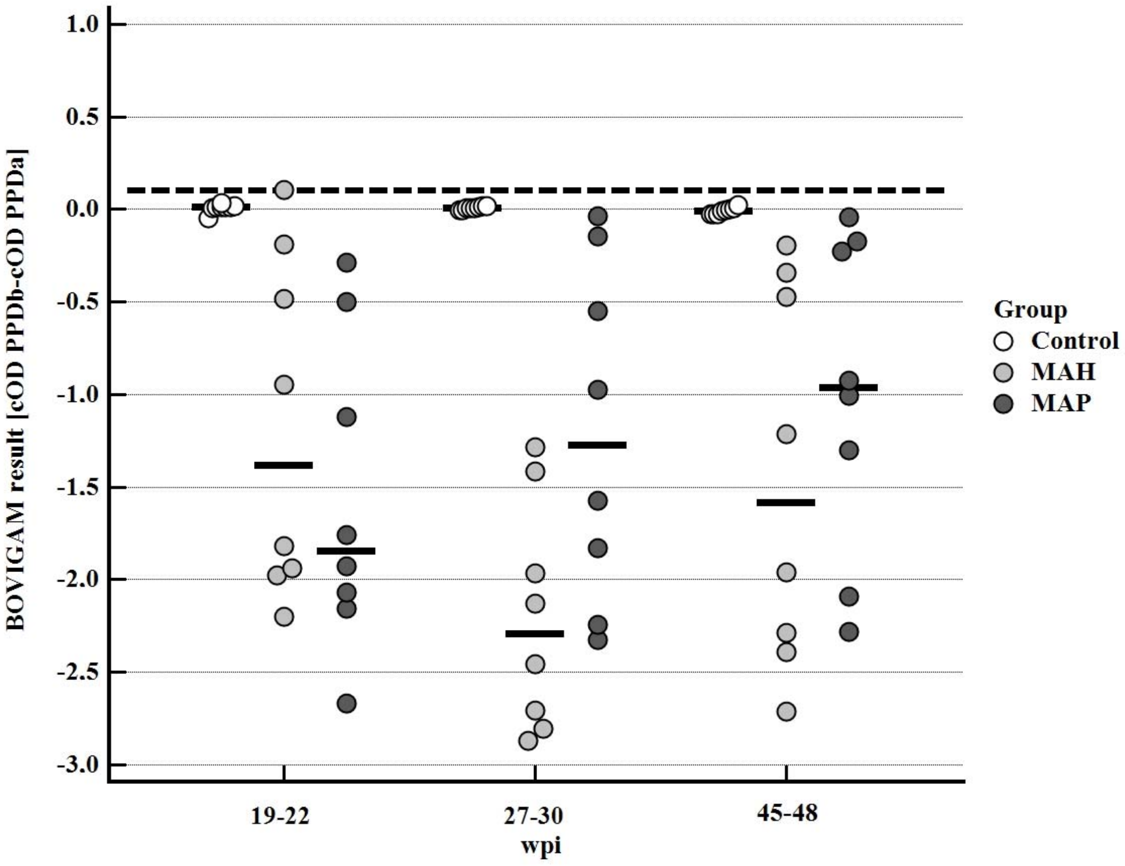

Figure S1: Difference of the whole blood IFN-γ response induced by bovine PPD (PPDb, 300 U) and avian PPD (PPDa, 250 U) of MAH or MAP inoculated goats versus control animals. Each dot represents one animal; bars represent medians. The dashed line indicates the lower limit of a positive response applying the analysis criteria of the BOVIGAM Test System [cOD PPDb-cOD PPDa].

Group	wpi	Goat No.	L5P	Hyd L5P	Map 1365	Map 0268c(it)	Map 0268c	Map 2872c	Map 4147	Map 1653	Map 1589c	Map 4000c	Map 0210c	Map 1693c	Map 2020	Map 3651cT(it)	Map 3651c
Control	19-22	45	-0,040	-0,025	-0,042	-0,031	0,050	-0,029	-0,013	-0,014	-0,004	-0,035	-0,033	0,007	-0,030	-0,019	-0,034
		46	-0,027	-0,034	0,085	0,123	0,466	0,277	0,556	0,933	0,837	0,421	0,039	0,208	0,233	0,244	0,319
		47	-0,045	-0,052	-0,044	-0,032	0,077	-0,023	0,070	0,143	0,123	-0,008	-0,046	-0,031	-0,024	-0,027	-0,034
		49	-0,030	-0,043	-0,030	-0,016	0,081	-0,008	0,009	0,078	0,025	-0,020	-0,039	-0,030	-0,021	-0,027	-0,016
		50	-0,022	-0,025	-0,020	-0,019	-0,010	-0,026	-0,014	0,009	0,004	-0,018	-0,021	-0,013	-0,013	-0,003	-0,018
		51	-0,003	-0,009	0,041	0,059	0,460	0,287	0,844	1,020	0,961	0,048	-0,004	0,063	0,067	0,125	0,166
		52	-0,003	-0,012	0,011	0,040	0,453	0,457	0,723	0,596	1,288	0,022	-0,001	0,053	0,088	0,073	0,094
		54	0,013	-0,005	0,001	0,035	0,074	0,040	0,145	0,346	0,263	0,031	-0,006	0,094	0,206	0,051	0,058
	27-30	45	-0,016	-0,021	-0,020	-0,016	0,002	-0,017	-0,007	0,000	-0,003	-0,014	-0,020	-0,006	-0,011	-0,014	-0,013
		46	-0,001	-0,007	0,016	0,037	0,100	0,077	0,104	0,197	0,137	0,072	0,000	0,030	0,040	0,036	0,020
		47	-0,091	-0,093	-0,093	-0,033	0,082	-0,082	-0,082	0,219	0,125	0,101	-0,080	-0,076	-0,047	-0,083	-0,071
		49	-0,004	-0,008	-0,006	0,011	0,016	-0,002	-0,002	0,063	0,052	0,003	-0,005	-0,004	0,024	-0,001	-0,004
		50	-0,030	-0,035	-0,033	-0,030	-0,024	-0,032	-0,032	-0,030	-0,020	-0,031	-0,033	-0,032	-0,029	-0,032	-0,032
		51	-0,016	-0,031	0,049	0,131	0,477	0,026	0,026	0,469	0,579	0,103	-0,026	0,090	0,143	0,063	0,210
		52	-0,012	-0,015	-0,014	-0,004	0,068	0,009	0,009	0,156	0,106	0,001	-0,013	0,001	0,008	0,018	-0,014
		54	-0,016	-0,020	0,025	0,085	0,233	0,069	0,069	0,664	0,495	0,015	-0,012	0,007	0,049	0,190	0,021
	45-48	45	-0,013	-0,005	-0,022	-0,016	0,121	0,005	0,005	-0,017	0,006	-0,020	-0,006	0,036	0,034	-0,014	-0,008
		46	0,006	0,001	0,008	0,021	0,059	0,010	0,010	0,134	0,080	0,011	0,002	0,009	0,015	0,034	0,030
		47	-0,028	-0,031	-0,009	0,014	0,143	-0,004	-0,004	0,261	0,152	0,144	-0,021	0,018	0,035	0,031	0,032
		49	0,008	0,056	-0,004	-0,009	0,038	0,003	0,003	0,020	0,039	-0,004	0,037	0,029	0,007	0,011	0,005
		50	-0,044	-0,053	-0,050	-0,034	0,006	0,016	0,016	-0,042	-0,002	-0,049	-0,030	-0,023	-0,040	-0,036	-0,048
		51	-0,011	-0,013	0,002	0,034	0,227	0,022	0,022	0,375	0,324	0,029	0,002	0,021	0,013	0,012	0,024
		52	0,014	-0,001	0,014	0,127	0,070	0,106	0,106	0,055	0,062	0,016	0,013	0,026	0,011	0,016	0,028
		54	0,003	-0,009	0,005	0,017	0,048	0,000	0,000	0,045	0,079	0,008	0,007	0,002	0,033	0,003	0,005
MAH	19-22	24	-0,022	-0,019	0,004	0,011	0,009	-0,007	-0,017	0,029	0,150	-0,011	-0,015	0,004	-0,002	0,000	0,039
		29	-0,084	-0,091	-0,082	-0,067	-0,035	-0,081	-0,070	-0,035	0,017	-0,085	-0,033	-0,058	0,123	-0,072	-0,089
		33	0,007	-0,014	0,012	0,006	0,069	0,017	0,092	0,183	0,313	0,025	0,040	0,010	0,283	0,006	0,026
		36	0,015	-0,020	0,000	-0,005	0,089	0,086	0,142	0,453	0,468	0,017	0,019	0,038	-0,004	0,001	0,009
		39	-0,019	-0,031	-0,017	-0,017	0,080	-0,021	0,065	0,069	0,669	-0,003	0,093	0,000	0,019	-0,011	0,051
		41	-0,040	-0,042	-0,022	-0,035	0,020	-0,036	-0,025	0,227	0,066	-0,003	-0,020	-0,019	0,036	-0,026	-0,008
		27	-0,015	-0,005	-0,003	-0,004	-0,003	-0,011	0,042	-0,016	0,011	-0,013	-0,010	-0,009	-0,015	0,050	0,057
		34	-0,016	-0,018	0,124	0,245	0,729	0,653	0,920	1,193	1,707	0,945	0,402	0,407	0,808	0,312	0,271
	27-30	24	0,001	0,012	0,001	-0,004	0,022	-0,004	0,001	0,126	0,136	-0,007	0,007	0,074	-0,007	0,153	0,063
		29	-0,001	0,013	0,009	0,177	0,064	0,012	0,028	0,029	0,070	0,001	0,018	0,045	0,087	0,094	0,019
		33	-0,011	0,007	0,020	0,001	0,036	0,019	0,019	0,027	0,077	0,057	0,028	0,000	0,404	-0,008	0,025
		36	-0,019	-0,024	0,043	0,002	0,080	-0,006	-0,006	0,437	0,388	0,039	0,022	-0,006	-0,010	-0,008	0,038
		39	-0,003	-0,008	0,012	0,026	0,083	-0,005	-0,005	0,130	0,304	0,061	-0,004	0,028	0,042	0,026	0,129
		41	-0,012	-0,019	0,002	0,002	0,017	0,068	0,068	0,211	0,147	-0,008	-0,014	0,010	0,022	0,028	0,038
		27	-0,008	-0,013	-0,004	-0,010	0,004	0,003	0,003	0,002	0,034	-0,013	-0,007	-0,005	-0,007	0,000	0,051
		34	-0,054	-0,016	0,079	0,176	0,502	-0,043	-0,043	0,887	0,828	0,228	0,139	0,041	0,137	0,133	0,106
	45-48	24	-0,057	-0,054	-0,059	-0,051	-0,028	-0,043	-0,043	-0,055	0,092	-0,059	-0,054	-0,030	0,016	-0,044	-0,011
		29	-0,002	0,042	-0,035	-0,034	0,046	-0,008	-0,008	-0,004	-0,006	-0,033	-0,015	-0,013	0,007	-0,013	-0,030
		33	-0,048	-0,046	-0,047	0,030	0,015	0,022	0,022	-0,004	0,009	-0,041	-0,017	0,001	0,065	-0,037	-0,047
		36	-0,086	-0,090	-0,063	0,063	0,131	-0,068	-0,068	0,269	0,214	0,005	-0,030	-0,027	-0,061	-0,054	-0,010
		39	-0,007	-0,021	-0,017	-0,014	0,066	-0,005	-0,005	-0,012	-0,001	-0,018	-0,007	-0,014	-0,014	-0,016	-0,010
		41	-0,028	-0,030	-0,010	-0,024	0,162	0,004	0,004	0,016	0,047	-0,023	0,009	-0,006	-0,018	-0,010	-0,017
		27	0,005	-0,002	0,011	-0,013	0,021	0,013	0,013	-0,015	0,011	-0,013	0,006	-0,002	-0,002	-0,004	0,000
		34	-0,002	-0,013	0,044	0,134	0,407	0,003	0,003	0,499	0,570	0,280	0,093	0,115	0,127	0,169	0,132
MAP	19-22	5	-0,028	-0,037	-0,018	-0,012	0,489	0,015	0,084	0,186	0,279	0,079	0,089	0,004	0,004	0,024	0,153
		7	-0,034	-0,039	-0,030	-0,031	0,014	-0,021	0,039	0,134	0,128	-0,010	0,030	-0,006	-0,026	-0,001	0,028
		11	0,011	0,016	0,031	0,028	0,091	0,031	0,084	0,320	1,110	0,058	0,093	0,059	0,371	0,394	1,202
		12	-0,052	-0,059	-0,047	-0,048	-0,012	-0,033	0,008	0,082	0,131	-0,013	0,020	-0,037	0,012	-0,001	0,030
		16	0,047	-0,027	-0,013	0,111	0,056	-0,017	-0,003	0,125	0,059	0,010	0,005	0,023	0,179	0,018	1,299
		20	-0,164	0,016	-0,149	-0,135	-0,103	-0,141	-0,124	1,046	-0,110	0,355	0,818	1,378	2,855	0,822	1,675
		21	-0,001	0,023	0,002	-0,006	0,056	-0,016	0,026	0,109	0,008	0,044	0,134	0,158	0,149	0,269	2,146
		23	-0,001	0,094	0,014	0,028	0,062	0,020	0,065	1,222	0,271	0,550	0,313	0,389	0,525	1,143	3,058
	27-30	5	0,036	0,046	0,085	0,086	0,269	0,180	0,430	0,409	0,781	0,312	0,629	0,122	0,112	0,409	0,944
		7	-0,010	-0,025	-0,017	-0,012	-0,011	-0,020	-0,011	-0,002	0,011	-0,017	0,063	-0,009	-0,014	-0,016	0,016
		11	-0,007	-0,019	-0,009	-0,011	0,071	-0,008	-0,008	0,204	0,224	0,008	-0,002	-0,006	0,053	0,015	0,067
		12	-0,025	-0,028	0,004	-0,017	0,028	0,021	0,021	0,048	0,116	-0,003	0,099	0,017	0,033	0,179	0,226
		16	0,024	0,025	-0,002	-0,010	0,007	-0,006	-0,006	0,003	-0,003	-0,017	-0,011	-0,014	0,009	-0,006	0,296
		20	-0,123	-0,138	-0,105	-0,074	-0,054	-0,090	-0,090	2,999	-0,030	0,832	1,981	2,576	3,789	2,682	3,702
		21	-0,013	0,025	-0,001	-0,018	0,006	0,003	0,003	0,122	0,005	0,053	0,052	0,044	0,046	0,173	1,391
		23	-0,001	0,069	0,053	0,027	0,046	-0,013	-0,013	0,838	0,190	0,537	0,770	0,837	0,911	1,653	3,802
	45-48	5	0,015	0,003	0,020	0,000	0,056	0,046	0,046	0,001	0,041	0,002	0,055	0,012	0,002	0,116	0,137
		7	-0,015	-0,004	-0,026	-0,017	0,066	-0,002	-0,002	0,035	0,056	-0,007	0,062	0,011	-0,007	0,017	0,010
		11	0,008	0,202	0,001	-0,004	0,027	0,003	0,003	0,173	0,543	0,041	0,261	0,029	1,489	1,037	2,677
		12	0,026	-0,027	-0,020	-0,032	0,023	-0,002	-0,002	-0,003	0,028	-0,024	-0,005	0,015	-0,010	0,000	0,028
		16	0,033	-0,008	-0,001	0,005	0,012	0,053	0,053	0,001	0,005	-0,006	-0,002	-0,003	0,017	-0,005	0,098
		20	-0,024	-0,005	-0,005	-0,012	0,038	-0,002	-0,002	0,230	0,091	0,104	0,276	0,342	0,504	0,197	0,317
		21	-0,017	-0,023	-0,034	-0,030	0,004	0,011	0,011	-0,015	0,002	-0,029	-0,014	-0,012	0,016	0,004	0,091
		23	0,016	0,037	0,015	0,008	0,068	0,007	0,007	0,118	0,109	0,105	0,042	0,201	0,118	0,174	0,753

S-N negative 0 positive

Figure S2: Heatmap representing the individual IFN-γ responses [S-N] of control, MAH and MAP inoculated goats after whole-blood stimulation with putatively MAP-specific antigens at the three testing time points.
